# The Art of Diagnosis

**Published:** 1919-08-09

**Authors:** 


### The Hospital

The Workers' Newspaper of Administrative Medicine and Institutional Life, Administration, National Insurance and Health.

No. 1731, Vol. LXVI. SATURDAY, AUGUST 9, 1919. PRICE TWOPENCE

### THE ART OF DIAGNOSIS.

It is impossible to consult any modern medical journal without encountering repeated references to novelty and improvement in the long list of experimental scientific phenomena applied to the diagnosis of disease. Not alone in this country, but throughout every centre of medical teaching in the world, this aspect of medical work is coming more and more into prominence, and there are those who would have us believe that in mankind's fight against disease, or, more accurately, the very beginnings of disease, there are two factors of paramount importance, namely, the medico-scientific specialist and the verdicts from his laboratory. And true it is that the work of this branch of the profession has already been invaluable to both armies and civil communities, and that many of the discoveries which have come to us through its agency have marked out the present time as the commencement of a new era in medicine.

But, as with all innovations, there inevitably arises an initial tendency to exaggerate, to over-apply, and to concentrate too great a responsibility upon the shoulders of a so promising assistant. In the future, laboratory research will undoubtedly lead to yet more perfect disease control, and this attractive prospect, the lure of the unknown and the experimental, is taking increasing numbers of the younger generation into laboratory service; but, valuable though their work will be, we must remember that, however cleverly and accurately their reports may remove difficulties and conundrums of diagnosis, their efforts can only reach a maximum utility when made in conjunction with an expert clinician. That is, an expert comparable with men of that magnificent school of physicians who had already achieved fame and success when laboratory investigation was in its infancy.

There are many instances of disease, the diagnosis of which can be instantly made after receipt of a laboratory report; in others, the indications are far more indefinite, and, though the matured clinicians of the present day undoubtedly realise the exact position and the limitations on both sides, yet there is, we believe, a noticeably definite risk, that unless these men make special note in their teaching, their younger followers and pupils will become daily more apt to lose sight of the enormous importance of bedside diagnosis, unaided by any modern scientific adjuncts, of either mechanical or laboratory origin. An extremely small percentage of the country's doctors have the advantage of possible prompt co-operation with an investigating centre, and, if this danger be not averted, the younger men must inevitably, when going out into general practice, find the whole backbone of their diagnostic principles wanting.

To the matured general practitioner himself such remarks may appear very obvious, but his training was at least founded by the old school of teaching, and the country may well be proud of his present-day efficiency. Here we refer to the future, to the men who will naturally succeed him, but who will be trained in the reformed medical schools which are proposed among the many post-war reconstructions. To these hospitals and schools we would urge that, however perfectly developed are their schemes of co-ordination, elaboration of special departments, and extension of accessory investigational units, they should take the greatest care that the finer points of diagnosis, the points of the old school, be not neglected. No less important is detailed teaching of psychological medicine as applied to every patient, rather than merely to those visited in mental hospitals, and this we believe to be a most necessary reform in the modern curriculum.

There is no need here to write an appreciation of the diagnostic skill of the physicians of two decades ago, their achievements and reputation will live on. It is our object simply to point out the need that modern advantages must be combined with those which that learning of these now elderly gentlemen have handed on to us. If we fail correctly to co-ordinate the true value of the new sciences with that of unaided clinical work, then the future may produce, not universal medical degeneration perhaps, but at least the regrettably frequent appearance of certain most unsatisfactory types of medical thought. The freak diagnosis, the exaggeration of unimportant pathological states with neglect of the vital condition, and many others are all in imminent danger of being overdone.

Apart from the discredit which may accrue to consultants, if this tendency continues, there is the certain fact that with yet more public medical education and enlightenment, the patients of the general practitioner will lose considerably in confidence if all his work must be guided by laboratory investigations, without which he appears to be helpless. The Press tends for the moment to urge the advantages, direct and immediate, of application of science to every patient; but we believe that the public must and should revert to correctly proportioned appreciation of the true clinician, to the practitioner who has studied and has not lost the true art of the old-school diagnosis.

